# Trends in mortality and life expectancy in Fiji over 20 years

**DOI:** 10.1186/s12889-021-11186-w

**Published:** 2021-06-22

**Authors:** Catherine Dearie, Christine Linhart, Eric Rafai, Devina Nand, Stephen Morrell, Richard Taylor

**Affiliations:** 1 School of Population Health, University of New South Wales, Samuels Building, Botany St, UNSW Sydney, NSW 2052 Australia; 2 Ministry of Health and Medical Services (MoHMS), Government of Fiji, Suva, Fiji

**Keywords:** Infant mortality, Under-5 mortality, Life expectancy, Fiji, Trend analysis, Decomposition

## Abstract

**Background:**

Fiji, a Pacific Island nation of 884,887 (2017 census), has experienced a prolonged epidemiological transition. This study examines trends in mortality and life expectancy (LE) in Fiji by sex and ethnicity over 1996–2017, with comparisons to published estimates.

**Methods:**

Trends in infant mortality rates (IMR), under-5 mortality (U5M), adult mortality (probability of dying), LE (at birth) and directly age-standardised death rates (DASRs) by sex and ethnicity, are calculated (with 95% confidence limits) using unit death records from the Fiji Ministry of Health and Medical Services. The LE gap between populations, or within populations over time, is examined using decomposition by age. Period trends are assessed for statistical significance using linear regression.

**Results:**

Over 1996–98 to 2014–17: IMR and U5M for i-Taukei and Fijians of Indian descent declined; U5M decline for i-Taukei (24.6 to 20.1/1000 live births) was significant (*p* = 0.016). Mortality (15–59 years) for i-Taukei males was unchanged at 27% but declined for Indians 33 to 30% (*p* = 0.101). Mortality for i-Taukei females increased 22 to 24% (*p* = 0.011) but declined for Indians 20 to 18% (*p* = 0.240). DASRs 1996–2017 were lower for i-Taukei (9.3 to 8.2/1000 population) than Indian males (10.6 to 9.8/1000). DASRs declined for i-Taukei (both sexes, *p* < 0.05), and for Indians (both sexes, *p* > 0.05). Over 22 years, LE at birth increased by 1 year or less (*p* = 0.030 in male i-Taukei). In 2014–17, LE (years) for males was: i-Taukei 64.9, Indians 63.5; and females: i-Taukei 67.0 and Indians 68.2. Mortality changes in most 5-year age groups increased or decreased the LE gap less than 10 weeks over 22 years. Compared to international agency reports, 2014–17 empirical LE estimates (males 64.7, females 67.8) were lower, as was IMR.

**Conclusions:**

Based on empirical data, LE in Fiji has minimally improved over 1996–2017, and is lower than some international agencies report. Adult mortality was higher in Indian than i-Taukei men, and higher in i-Taukei than Indian women. Exclusion of stillbirths resulted in IMRs lower than previously reported. Differing mortality trends in subgroups highlight the need to collect census and health data by ethnicity and sex, to monitor health outcomes and inform resource allocation.

## Background

Comprehensive, timely measures of mortality and accurate assignment of causes of death are fundamental in understanding the health of a population and effectively allocating health resources [1]. Measures of mortality may be calculated directly from data collected and collated locally, with corrections for biases, or from modelling techniques [2]. Results are most accurate if based on complete local deaths and denominators from recently measured populations.

Fiji has experienced demographic and epidemiological transitions since the mid-twentieth century, with reductions in infectious diseases leading to mortality decline, increased life expectancy (LE) and population growth, followed by fertility reduction. Increased proportional mortality from cardiovascular and other non-communicable diseases (NCDs) in adults is considered to have contributed to the plateau in LE in Fiji since the mid-1980s [3, 4].

Fiji is the second most populous of the Pacific Island countries (PICs) in Oceania, with a population of 884,887 at the 2017 census, of which 81% resides on Viti Levu and 15% on Vanua Levu [5]. The proportion of the population aged > 65-years grew from 3 to 6% over 1996–2017 [5, 6]. The predominant ethnic groups in Fiji are: 62% i-Taukei (Melanesian) Fijians; and 31% Fijians of Indian descent (referred hereinafter as ‘Indians’); with 7% other Pacific Islanders, Asians, Europeans, and others [7].

Free, or low cost, public health care, funded through taxation, is provided by: three divisional (tertiary care), two specialist and 17 sub-divisional hospitals; 84 health centres (with a medical officer or nurse practitioner); and 98 nursing stations [8]. Government funding of health services in Fiji (2011) was amongst the lowest of PICs at 2.9–3.5% of gross domestic product [9]. Compared to the minimum World Health Organisation (WHO) standard (228 health workers/100,000 people), 9 of 15 provinces in Fiji were below target for nurses and none met the target for doctors in 2007 [10].

Cross-sectional studies have shown that Indians have a higher prevalence of hypertension [11] and type 2 diabetes mellitus (T2DM) at a lower body mass index (BMI) than i-Taukei [12]. Prevalence of obesity, and incidence and prevalence of T2DM [12, 13] increased over 1980–2011 in both sexes and major ethnicities (*p* < 0.05), with likely impact on premature mortality and LE [12]. Public health initiatives targeting NCDs have been implemented, following development of a *National NCD Strategic Plan 2015–19* [14]. Accurate and timely mortality measures based on local empirical data are required to monitor the success of these initiatives.

The *Fiji Births, Deaths and Marriages (BDM) Registration Act 1975* requires registration of every birth, and the completion of a *Medical Certificate of Cause of Death* (MCCD) by a medical or nurse practitioner or midwife, in accordance with the International Classification of Diseases (ICD) [15]. Every *Notification of Birth* (NoB) and MCCD is forwarded to the Data Analysis Management Unit (DAMU) of the MoHMS for collation. No fee is levied for timely registration of births or deaths, however the requirement for in-person attendance by a family member at the BDM office to complete the legal registration process may create a barrier to completion. DAMU birth and death records have been assessed as more complete than the civil register [16, 17].

Infant mortality rates (IMR), under-5 mortality (U5M) and adult mortality (15–59 years), as well as LE at birth, are widely reported epidemiological measures of population health. LE is included in the United Nations (UN) Human Development Index, [14] and is normally derived from age-specific death rates. Adult mortality is defined by the UN as the probability of dying between ages 15–60 years, [18] often considered as working ages, where mortality contributes to lost productivity. The causes of death are quite different between the two halves of the adult age range, and consequently examination of mortality in 15–34- and 35–59-year age ranges will better inform targeting of preventative health interventions. The gap, or lack thereof, in LE between populations, or over time within a population, is the sum of positive and negative contributions of age-specific mortality [19]. Decomposition of the LE gap can identify specific age groups or segments of the population as targets for mortality reduction interventions.

LE at birth, U5M and adult mortality from NCD’s at ages 30–69 years are included in the Healthy Island indicators adopted in *The Pacific Island Countries and Areas - WHO Cooperation Strategy 2018–2022* [8]. When accurately measured, these indicators facilitate monitoring of progress towards Sustainable Development Goal (SDG) three: ensuring healthy lives and well-being at all ages. By 2030, SDG 3.2. is to reduce newborn mortality to ≤12/1000 live births and to reduce U5M to ≤25/1000 live births; and SDG 3.4 is to reduce by one third premature mortality from NCDs [20].

This study estimates age-specific all-cause mortality and LE trends by ethnicity and sex in Fiji over 1996–2017 to assess the extent to which the previously reported plateaux in LEs during 1985–2007 [3] has persisted, and compares these estimates with published data from other sources. Age-specific mortality rate contributions to LE are also assessed.

## Methods

### Study design

In this population-based study, mortality rates by age group, sex and ethnicity are calculated to estimate adult mortality and LE using the hypothetical cohort method [21]. Infant and < 5 years deaths, and births in the same period, are used for IMR and U5M [22].

### Deaths

All deaths occurring in Fiji between January 1996 and December 2017 and reported to the Fiji MoHMS were analysed according to date of death (DoD) (including deaths recorded in 2018 occurring in 2017). De-identified unit records were extracted from the electronic recording systems maintained by the DAMU of MoHMS (Table 1), each record containing age at death or date of birth (DoB), DoD, cause of death, national health number (NHN), sex and ethnicity of the decedent. Whilst the tools used for recording details of deaths changed during 1996–2017, the process for reporting deaths did not. Electronic mortality records were maintained in a Microsoft Access database from 1996 to 2010, and in the mortality module of the Patient Information System (PATIS Plus) from 2012 onwards. The NHN is a unique identification number used to track and record any interaction of a person with the health system in Fiji, and can be used for confirmation of demographic information, such as age, sex and ethnicity during data entry from the MCCD into electronic systems.

**Table 1:** Number of deaths by sex, summarised by age groups, per triennia^b^, i-Taukei and Indian, Fiji 1996–2017

**Age**	**Deaths Male Total**^**a**^							**Deaths Female Total**^**a**^						
**Period**	**96–98**	**99–01**	**02–04**	**05–07**	**08–10**	**11–13**	**14–17**^b^	**96–98**	**99–01**	**02–04**	**05–07**	**08–10**	**11–13**	**14–17**^b^
< 1	509	436	500	591	471	501	734	438	369	425	502	352	434	498
1–4	171	201	154	160	246	172	189	140	173	119	124	202	120	153
5–14	207	238	188	178	150	139	175	136	187	162	118	105	99	142
15–34	747	819	788	787	681	778	967	494	577	601	518	528	498	707
35–59	2924	3359	3263	3581	3561	3788	5300	1920	1988	2182	2373	2572	2621	3800
60–74	2496	2948	3166	3309	3399	3813	5668	1848	2242	2479	2539	2957	3057	4463
≥75	1644	1891	1873	1795	1772	2011	2857	1588	1731	1834	1792	2037	2160	3372
Unk^c^	79	221	153	27	1	31	0	82	170	133	12	1	32	0
Total	8777	10,113	10,085	10,428	10,281	11,233	15,890	6646	7437	7935	7978	8754	9021	13,135
	**Deaths i-Taukei Male**							**Deaths i-Taukei Female**						
< 1	322	287	339	407	336	371	567	268	256	303	345	244	335	378
1–4	128	155	120	119	191	135	161	113	142	89	102	163	95	122
5–14	115	143	116	124	90	106	129	82	95	106	77	74	64	100
15–34	375	390	382	423	380	435	520	261	305	333	314	323	294	449
35–59	1258	1401	1465	1741	1737	1941	2863	998	1122	1233	1431	1539	1641	2444
60–74	1325	1600	1762	1818	1873	2089	3175	1029	1316	1467	1508	1680	1666	2529
≥75	887	1019	1056	1014	1030	1148	1691	920	1031	1126	1091	1223	1249	1933
Unk^c^	41	100	81	12	1	17	0	49	99	84	7	1	21	0
Total	4451	5095	5321	5658	5638	6242	9106	3720	4366	4741	4875	5247	5365	7955
	**Deaths Indian Male**							**Deaths Indian Female**						
< 1	167	130	142	165	119	105	136	156	102	109	139	95	88	104
1–4	30	41	28	34	37	28	21	26	23	23	18	33	21	24
5–14	83	80	58	51	56	25	41	48	79	47	37	29	28	35
15–34	332	379	366	320	246	294	378	215	253	246	176	179	175	233
35–59	1528	1830	1637	1681	1643	1676	2205	855	790	855	845	932	891	1191
60–74	1050	1226	1246	1346	1367	1550	2251	752	856	930	934	1185	1285	1769
≥75	656	762	716	680	638	738	989	592	608	612	621	708	824	1285
Unk^c^	33	105	63	15	0	12	0	28	62	48	3	0	9	0
Total	3879	4553	4256	4292	4106	4428	6021	2672	2773	2870	2773	3161	3321	4641

^a^ Total includes i-Taukei, Indians, others. ^b^2014–17 4-year data. ^c^ Unk: deaths with unknown age proportionately redistributed prior to analysis

During the 1990s a separate form was used for recording stillbirths**,** but its use was discontinued in 2000. A new MCCD format from 2009 introduced recording of DoB and added a checkbox to identify stillbirths. Up to 172 foetal deaths (ICD-10 P95) per year (0–2.5% of total deaths) were identified in the unit death records for 2000–17, with higher numbers found in the data extracted from PATIS Plus from 2012 to 17 (160–172 per year). These deaths, clearly identified as stillbirths (P95, DoB and DoD identical), were removed prior to all analyses.

Deaths with unknown age were proportionately redistributed. These comprised 2.2% of total deaths in 1999–2001 and 0.3–1.6% in other periods. Annual numbers of deaths in the MoHMS data compared with those published by Fiji Bureau of Statistics (FBoS) for 1996–2008, [17] and WHO for 1996–2012, [23] matched exactly for most years. MoHMS data differed for 4 years by 8–51 deaths/year, representing < 1% of deaths, but were higher than WHO data by 3.5% (203) in 2001 and 10% (601) in 2003. Differences are likely a result of supply of mortality data to agencies prior to complete enumeration by Fiji MoHMS, and/or inclusion of stillbirths in counts of infant deaths. During June–December 2018 an additional 348 deaths were recorded in the MoHMS database with a DoD between January–December 2017; mortality data from earlier years were essentially complete at the time of extraction in June 2018.

### Deduplication

Records with the same death certificate number, DoD, DoB, cause of death, sex and ethnicity were considered duplicates and were removed. The highest number of duplicated records occurred in 2017; de-duplication reduced the deaths by 0.3%. Previous reviews of Fiji mortality concluded MoHMS death data as the most reliable source for mortality analysis from 1996, [14] and death registration, by sex and ethnicity, for 1996–2004 was assessed as essentially complete (≥95% excepting females 2002–04 at 85%), [3, 4, 17] using the Brass growth-balance method [24].

### Populations and births

Population denominators are from Fiji Censuses. Populations by ethnicity (by 5-year age group and sex) were published by the FBoS following the 1996 [6] and 2007 Fiji Censuses [25]. The 2017 census population (884,887 compared to 837,271 at the 2007 census) was lower than projections from 2007 for 2017 (~ 910,000) [26]. Ethnicity data were not released following the 2017 census because of reservations concerning data quality [27]. Ethnicity projections published by FBoS in 2017 [7] were used to estimate the ethnic composition of the 2017 population; this projected an increase in i-Taukei, as a proportion of the total population, from 56.8 to 62.1%, and a decrease in Indians from 37.5 to 30.7% [7]. The proportion of each ethnic group in 2007 in each 5-year age group, by sex, was multiplied by the ratio of FBoS projected population proportions (%) (62.1/56.8 for i-Taukei and 30.7/37.5 for Indian) to estimate the populations by ethnicity for 2017. Annual populations for intercensal years, by sex and five-year age group, from 0 to 4 years to ≥75 years, were estimated by linear interpolation of Fiji populations from the 1996, [6] 2007 [25] and 2017 censuses [5].

Data on live births per year were available from the MoHMS for 1996–2017 by ethnicity, but not by sex. In 2012–17, 99.5% of births are reported to have occurred within health facilities, [16] supporting the reliability of birth statistics.

### Analysis

To reduce variation from small event numbers and enumeration biases, live births, deaths, and populations over 1996–2013 were grouped by triennia, with the most recent period containing four-years (2014–17) to avoid the potential of stochastic variation from analysis of single-year data.

All-cause mortality was estimated as IMR and U5M; adult mortality, expressed as the probability (%) of dying over 15–34 years (young adult, _20_q_15_), 35–59 years (mid-age adult, _25_q_35_), 15–59 years (adult, _45_q_15_) and 30–69 years (_40_q_30_), [8]; and LE at birth. There is some overlap of mortality measures, consistent with overlaps in the currently monitored parameters by international agencies, such as Global Burden of Disease (GBD), WHO and UN. Comparisons involving LE (hypothetical cohort method) are not affected by differing age structures of populations. All-age directly age-standardised mortality rates (DASRs) were calculated using the 2007 Fiji census population as the standard, in 5-year age groups (< 5 to ≥75 years).

IMR was calculated using deaths < 1-year divided by live births in the same period (_1_q_0_), and U5M (_5_q_0_) calculated from deaths < 5-years divided by live births in the same period [22]. For sex specific lifetables, mortality < 5 years was estimated using population denominators. Adult mortality was calculated from the sum of mortality rates (*m*_*x*_) in each 5-year age group (*n*_*x*_) over the broader age interval (cumulative rate), with the probability of dying in the specified age interval (cumulative risk) derived as: $Cumulative\ rate={\sum}_{x= first\  age\  group}^{last\  age\  group}\left({m}_x{n}_{x.}\right)$

*Cumulative risk* = 1 − *e*^(*cumulative rate*)^ [28].

All mortality estimates, 95% confidence intervals (CIs) (normal approximation of the binomial) and CIs for LE (derived from the Chiang II method for estimating variance of the probability of dying) [21] were calculated using Microsoft Excel; the calculation tools, with explanatory notes, are publicly available [29].

LE differences between 1996 and 98 and 2014–17, and between ethnic groups by sex, were decomposed to examine the contribution of mortality by 5-year age groups to LE gaps, following Arriaga’s method, [20] using Excel spreadsheets adapted from those developed by Auger et al. [30]

**Fig. 1:**
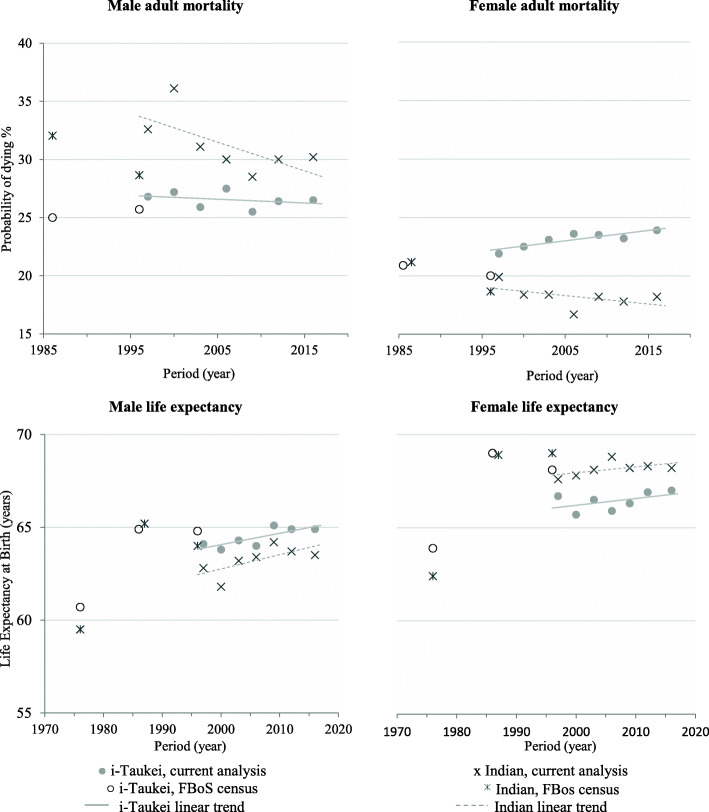
Adult (15–59 years) risk of mortality and life expectancy, by ethnicity, Fiji 1975–2017. 1976, Census survivorship data used to impute model life tables [31]. 1986, Child and adult survival data used to impute logit model life tables [32]. 1996, Vital registration and census data used to impute model life tables (UN Far Eastern 2 parameter model) [6]. All other data from present analysis. FBoS: Fiji Bureau of Statistics

Linear regression was used to assess the statistical significance of period trends, by determining a *p*-value for trend for U5M, adult mortality, LE and DASR, using Proc Reg is SAS, Version9.4. Earlier estimates of adult mortality (probability of dying 15–59 years) and LE at birth, for both sexes and ethnicities, from indirect demographic methods from Fiji Censuses of 1976, 1986 and 1996, are displayed (Fig. 1) to show longer term trends.

### Comparisons

Published Fiji mortality estimates from diverse sources from 1963 to 2018 for IMR and U5M (Fig. 3), and LE by sex from 1960 to 2018 (Fig. 4) are displayed.

## Results

### Infant and under 5 mortality (Table 2)

An overall downward trend in IMR and U5M was observed over 1996–2017, however, IMR increased during 2002–07, and higher U5M was observed in 2005–07. The IMR stabilised during 2011–2017 at around 15/1000 live births annually for both major ethnicities. U5M was lower in Indians than i-Taukei, with a narrowing gap during 1996–2017. Sex-specific IMR and U5M were not calculated since live births by sex were not available from MoHMS.

**Table 2:** Infant mortality and under-five mortality rates, total, i-Taukei and Indian, Fiji 1996–2017

Period	i-Taukei			Indian			Total^**b**^		
**IMR**	**Births**	**IMR**	**95% CI**	**Births**	**IMR**	**95% CI**	**Births**	**IMR**	**95% CI**
1996–1998	11,272	17.5	16.1–18.9	6059	17.8	15.8–19.7	18,349	17.2	16.1–18.3
1999–2001	11,353	16.2	14.8–17.5	5026	15.4	13.4–17.4	17,310	15.9	14.8–17.0
2002–2004	11,870	18.0	16.6–19.4	4750	17.6	15.4–19.8	17,542	17.6	16.4–18.7
2005–2007	12,651	19.8	18.4–21.2	4884	20.7	18.4–23.0	18,506	19.7	18.5–20.8
2008–2010	14,222	13.6	12.5–14.7	4840	14.7	12.8–16.7	19,962	13.7	12.8–14.7
2011–2013	15,247	15.5	14.4–16.7	4381	14.7	12.6–16.7	20,524	15.2	14.3–16.2
2014–2017^a^	15,286	15.5	14.5–16.5	3757	16.0	14.0–18.0	19,896	15.5	14.6–16.3
**U5M**	**Births**	**U5M**	**95% CI**	**Births**	**U5M**	**95% CI**	**Births**	**U5M**	**95% CI**
1996–1998	11,272	24.6	23.0–26.2	6059	20.8	18.8–22.9	18,349	22.8	21.6–24.1
1999–2001	11,353	24.9	23.2–26.6	5026	20.5	18.2–22.8	17,310	23.1	21.8–24.4
2002–2004	11,870	23.9	22.3–25.5	4750	21.2	18.8–23.6	17,542	22.7	21.5–24.0
2005–2007	12,651	25.6	24.0–27.2	4884	24.3	21.8–26.8	18,506	24.8	23.5–26.9
2008–2010	14,222	21.9	20.5–23.3	4840	19.6	17.3–21.8	19,962	21.2	20.1–22.4
2011–2013	15,247	20.6	19.3–21.9	4381	18.4	16.1–20.7	20,524	20.0	18.9–21.1
2014–2017^a^	15,286	20.1	19.0–21.2	3757	19.0	16.8–21.1	19,896	19.8	18.8–20.8
p trend U5M		**0.016↓**			0.311			0.069	

Deaths corrected for removal of stillbirths and duplicates. ^a^2014–17 4 yr data. ^b^ Total include i-Taukei, Indians, others. Births: average per year. *IMR* infant mortality rate/1000 from deaths < 1 yr and live births, *U5M* under 5 yr mortality/1000 live births from deaths < 5 yrs. and live births. **Bold:** ***p*** **< 0.05**; *Italics*: *p* ≥ *0.05 to p < 0.06*. Births by sex not available. *CI* confidence interval

### Adult mortality (Table 3, Fig. 1)

Adult mortality across all age groups and by major ethnicity was significantly higher for males than females (non-overlapping 95% CIs). In young adults (15–34 years), a downward linear trend was evident for i-Taukei males (*p* = 0.040) over 1996–2017. The probability of dying for females 15–34 years remained low (around 3%) over the study period, with no significant difference between or discernible trend for i-Taukei (*p* = 0.237) and Indians (*p* = 0.481). Mortality was significantly higher for Indian men than i-Taukei men aged 35–59, 15–59 and 30–69 years over the whole study period (non-overlapping 95% CI). Mortality was lower in women aged 35–59, 15–59 and 30–69 years for Indian than i-Taukei over the whole study period (non-overlapping 95% CI’s, excepting for some 30–69-year mortality).

**Table 3:** Adult mortality, life expectancy, age standardised rates by age group, ethnicity, sex. Fiji 1996–2017

Period	Male						Female					
	i-Taukei		Indian		Total^**b**^		i-Taukei		Indian		Total^**b**^	
**Young adult mortality 15–34 years:** probability dying (%) and 95% CI												
1996–98	3.6	3.3–4.0	3.6	3.2–3.9	3.6	3.3–3.8	2.6	2.3–3.0	2.4	2.1–2.8	2.5	2.3–2.7
1999–01	3.7	3.3–4.0	4.1	3.7–4.5	3.9	3.6–4.2	2.9	2.6–3.2	2.9	2.6–3.3	2.9	2.6–3.1
2002–04	3.3	3.0–3.6	4.0	3.6–4.4	3.6	3.3–3.8	3.0	2.7–3.3	2.9	2.6–3.3	2.9	2.7–3.1
2005–07	3.4	3.1–3.8	3.5	3.1–3.8	3.4	3.2–3.7	2.6	2.4–2.9	2.1	1.8–2.4	2.4	2.2–2.6
2008–10	3.0	2.7–3.3	2.8	2.4–3.1	3.0	2.8–3.2	2.6	2.4–2.9	2.3	1.9–2.6	2.5	2.3–2.7
2011–13	3.4	3.1–3.7	3.6	3.2–4.0	3.5	3.2–3.7	2.4	2.1–2.6	2.4	2.0–2.7	2.4	2.2–2.6
2014–17^a^	3.0	2.8–3.3	3.7	3.4–4.1	3.3	3.1–3.5	2.6	2.4–2.9	2.5	2.2–2.9	2.5	2.3–2.7
p trend	**0.040↓**		0.470		0.134		0.237		0.481		0.270	
**Mid age mortality 35–59 years:** probability dying (%) and 95% CI												
1996–98	24.0	22.8–25.2	30.1	28.8–31.4	26.7	25.8–27.5	19.8	18.7–20.9	17.9	16.8–19.0	18.5	17.8–19.3
1999–01	24.4	23.3–25.5	33.3	32.0–34.6	28.1	27.3–29.0	20.2	19.1–21.3	15.9	14.9–16.9	17.9	17.1–18.6
2002–04	23.4	22.3–24.5	28.2	27.0–29.4	25.5	24.7–26.2	20.8	19.7–21.8	16.0	15.0–17.0	18.3	17.6–19.0
2005–07	24.9	23.9–25.9	27.5	26.3–28.6	25.7	25.0–26.4	21.6	20.5–22.6	14.9	13.9–15.8	18.2	17.5–18.8
2008–10	23.1	22.2–24.1	26.5	25.3–27.6	24.2	23.5–24.9	21.4	20.5–22.4	16.4	15.4–17.3	18.7	18.1–19.4
2011–13	23.8	22.9–24.7	27.4	26.3–28.6	24.6	23.9–25.3	21.3	20.4–22.2	15.8	14.9–16.8	18.3	17.6–18.9
2014–17^a^	24.2	23.4–24.9	27.5	26.5–28.4	24.5	23.9–25.1	21.8	21.1–22.6	16.0	15.2–16.9	18.7	18.2–19.3
p trend	0.830		0.086		**0.025↓**		**0.006↑**		0.304		0.259	
**Adult mortality 15–59 years:** probability dying (%) and 95% CI												
1996–98	26.8	25.6–28.0	32.6	31.3–33.9	29.3	28.5–30.2	21.9	20.8–23.0	19.9	18.8–21.1	20.6	19.8–21.3
1999–01	27.2	26.0–28.3	36.1	34.8–37.3	31.0	30.1–31.8	22.5	21.5–23.6	18.4	17.3–19.4	20.2	19.5–20.9
2002–04	25.9	24.9–27.0	31.1	29.9–32.3	28.1	27.4–28.9	23.1	22.1–24.2	18.4	17.4–19.5	20.7	20.2–21.4
2005–07	27.5	26.5–28.5	30.0	28.8–31.1	28.3	27.5–29.0	23.6	22.6–24.6	16.7	15.7–17.6	20.2	19.5–20.8
2008–10	25.5	24.5–26.4	28.5	27.4–29.6	26.5	25.8–27.2	23.5	22.5–24.5	18.2	17.3–19.2	20.8	20.1–21.4
2011–13	26.4	25.5–27.3	30.0	28.9–31.2	27.2	26.5–27.9	23.2	22.2–24.1	17.8	16.8–18.8	20.2	19.6–20.8
2014–17^a^	26.5	25.7–27.2	30.2	29.2–31.2	27.0	26.4–27.5	23.9	23.1–24.7	18.2	17.3–19.0	20.8	20.2–21.3
p trend	0.499		0.101		**0.030↓**		**0.011↑**		0.240		0.657	
**Adult mortality 30–69 years:** probability dying (%) and 95% CI												
1996–98	50.9	49.2–52.4	59.8	58.0–61.4	54.4	53.2–55.5	42.1	40.5–43.7	41.3	39.4–43.1	41.1	39.9–42.2
1999–01	52.4	50.9–53.9	60.6	59.0–62.1	55.4	54.4–56.5	45.0	43.4–46.4	39.3	37.6–41.0	41.7	40.6–42.8
2002–04	50.3	48.9–51.7	55.4	53.8–56.9	52.0	51.0–53.0	44.8	43.4–46.2	37.4	35.8–39.0	40.8	39.3–42.4
2005–07	50.5	49.2–51.8	54.1	52.6–55.6	51.5	50.5–52.4	44.1	42.7–45.4	35.6	34.0–37.1	39.7	38.7–40.7
2008–10	47.9	46.6–49.2	53.0	51.5–64.5	49.4	48.4–50.3	43.2	41.9–44.5	40.2	38.6–41.6	40.9	40.0–41.9
2011–13	48.3	47.0–49.5	55.6	54.1–57.0	50.1	49.2–51.0	41.7	40.5–42.9	40.1	38.6–41.6	39.7	38.7–40.6
2014–17^a^	49.4	48.4–50.4	57.6	56.3–58.8	51.0	50.2–51.7	42.4	41.4–43.4	39.9	38.6–41.2	39.9	39.1–40.7
p trend	*0.062***↓**		0.268		**0.029↓**		0.340		0.970		*0.061***↓**	
**Life expectancy (LE) at birth:** LE and 95% CI												
1996–98	64.1	63.7–64.5	62.8	62.4–63.2	63.6	63.4–63.9	66.7	66.4–67.1	67.6	67.2–68.1	67.4	67.1–67.6
1999–01	63.8	63.4–64.1	61.8	61.5–62.2	63.0	62.8–63.3	65.9	65.5–66.2	68.0	67.6–68.4	67.0	66.7–67.2
2002–04	64.3	64.0–64.6	63.2	62.8–63.6	63.9	63.6–64.1	65.9	65.5–66.2	68.2	67.8–68.6	67.0	66.8–67.3
2005–07	64.0	63.7–64.4	63.4	63.0–63.8	63.9	63.7–64.2	66.0	65.7–66.4	68.9	68.5–69.3	67.4	67.1–67.6
2008–10	65.1	64.7–65.4	64.2	63.8–64.6	64.8	64.5–65.0	66.4	66.0–66.7	68.2	67.8–68.6	67.4	67.1–67.6
2011–13	64.9	64.6–65.2	63.7	63.3–64.1	64.6	64.4–64.8	66.9	66.6–67.2	68.3	67.9–68.6	67.8	67.5–68.0
2014–17^a^	64.9	64.7–65.2	63.5	63.2–63.9	64.7	64.5–64.9	67.0	66.7–67.2	68.2	67.9–68.6	67.8	67.6–68.0
p trend	**0.030↑**		0.091		**0.014↑**		0.188		0.298		**0.048↑**	
**Direct age standardised death rates (DASR):** DASR and 95% CI												
1996–98	9.27	9.01–9.54	10.6	10.3–11.0	9.75	9.55–9.95	7.29	7.06–7.52	6.76	6.51–7.02	6.89	6.73–7.05
1999–01	9.49	9.23–9.74	11.4	11.1–11.7	10.1	10.0–10.3	7.69	7.47–7.92	6.37	6.14–6.61	6.97	6.82–7.13
2002–04	8.95	8.72–9.19	9.81	9.5–10.1	9.21	9.04–9.39	7.61	7.40–7.82	6.07	5.85–6.29	6.82	6.67–6.96
2005–07	8.65	8.43–8.87	9.23	8.96–9.50	8.73	8.57–8.90	7.21	7.01–7.41	5.48	5.28–5.68	6.34	6.20–6.48
2008–10	7.94	7.74–8.15	8.69	8.42–8.95	8.12	7.96–8.27	7.09	6.90–7.28	6.03	5.82–6.24	6.48	6.34–6.61
2011–13	8.17	7.97–8.37	9.49	9.21–9.76	8.47	8.31–8.62	6.64	6.46–6.82	6.27	6.05–6.48	6.25	6.12–6.38
2014–17^a^	8.23	8.06–8.40	9.79	9.55–10.0	8.50	8.37–8.63	6.72	6.57–6.87	6.50	6.32–6.69	6.35	6.25–6.46
p trend	**0.010↓**		0.143		**0.017↓**		**0.021↓**		0.718		**0.011↓**	

^a^ 2014–17 4-year data. ^b^ Total includes i-Taukei, Indians, others. **Bold:** ***p*** **< 0.05**; *Italics*: *p* ≥ *0.05 to p < 0.06*. *LE* life expectancy at birth, *CI* confidence interval, *DASR* all age, direct age standardised death rate per 1000 population based on Fiji 2007 census by 5-year age groups, *Probability dying (%)* probability of dying in the specified age interval = cumulative risk from the cumulative rate (see Methods)

Mid-age (35–59 years) male mortality remained unchanged for i-Taukei (*p* = 0.830) but declined slightly for Indians (*p* = 0.086) and declined for total males (*p* = 0.025). Mid-age female mortality increased for i-Taukei (*p* = 0.006) over 1996–2017 but was relatively unchanged for Indians (*p* = 0.304) and for total females (*p* = 0.259). Trends in the broader 15–59-years mortality for each ethnicity and sex are similar to those seen in 35–59 years mortality.

### Direct age standardised death rates and life expectancy at birth (Table 3, Fig. 1)

DASRs decreased over 1996–2017 for both ethnicities and sexes, with trends statistically significant for the total population by sex (M: *p* = 0.017, F: *p* = 0.011) and for i-Taukei (M: *p* = 0.010, F: *p* = 0.021), but not for Indians.

Over 1996–2017, LE at birth increased in i-Taukei males (0.8 years, *p* = 0.030) and females (0.3 years, *p* = 0.188), and Indian males (0.7 years, *p* = 0.091) and females (0.5 years, *p* = 0.298). For the total population, LE increased 1.1 years in males (*p* = 0.014) and 0.4 years in females (*p* = 0.048) over 1996–2017.

### Decomposition of the life expectancy gap (Fig. 2)

For males, all age groups except < 5 and 25–29 years contribute to a small increase in LE between 1996 and 98 and 2014–17. For females, the positive contributions to the LE improvement come from reduced mortality in most 5-year age groups but are offset by negative contributions in the 30–44- and 50–59-years age ranges, resulting in a smaller overall increase in LE compared to males. Most of the negative contribution to the overall LE change for females between 1996 and 98 and 2014–17 can be attributed to i-Taukei women in the 30–44- and 50–59-years age ranges, with mortality in the 65–74 years range for Indian women also negatively impacting the overall LE gain for women in Fiji.

**Fig. 2:**
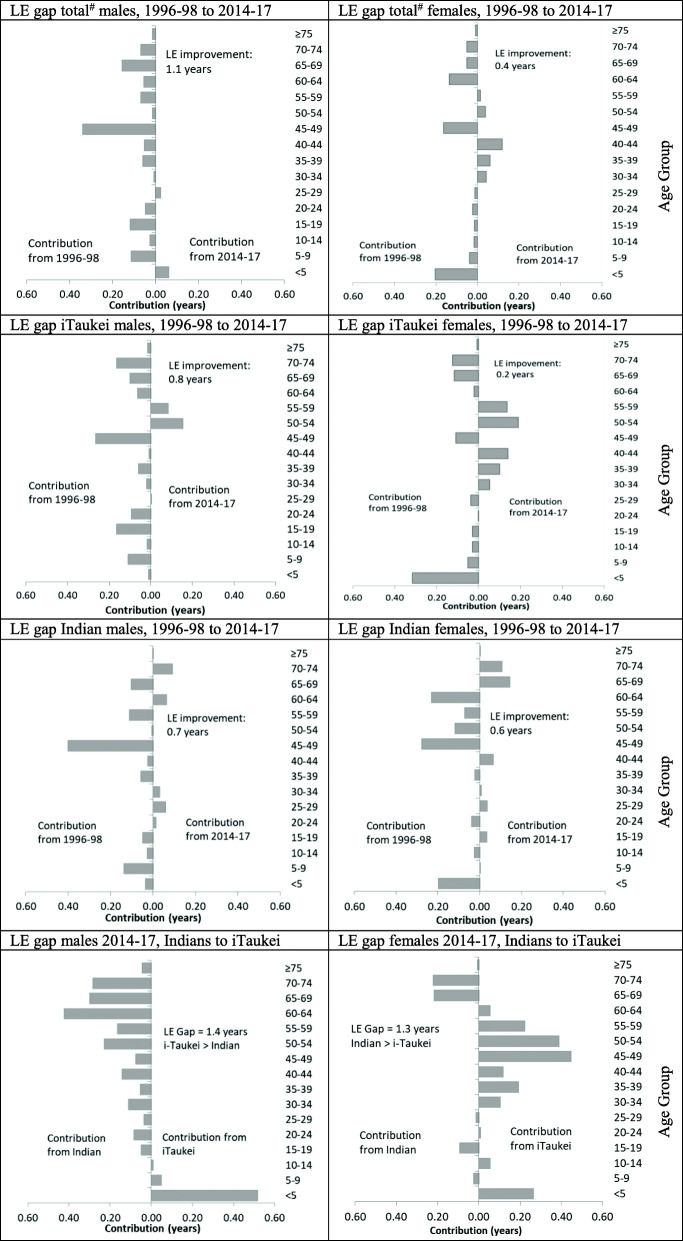
Decomposition of differences in life expectancy between 1996 and 98 and 2014–17, and between populations in 2014–17. # Total includes i-Taukei, Indian, others. LE: Life expectancy. Grey bars show the estimated LE deficit contributed by age-specific mortality of each 5-year age group to the LE gap between populations. Each panel displays the decomposition of the LE gap between the first entity and the second entity named (by convention)

For males in 2014–17, higher mortality in Indians in all age groups > 15 years contributes positively to the LE gap between i-Taukei and Indians, offset somewhat by the higher U5M in i-Taukei. For females in 2014–17, higher mortality in < 5- and 30–64-years in i-Taukei contributes positively to the LE gap but is partially offset by higher 65–74-years mortality in Indians, reducing the LE gap. Higher mortality in every 5-year-age group in the male population contributes to the LE gap between the sexes (not shown in Fig. 2).

### Comparison with mortality trends from other sources (Figs. 3 and 4)

**Fig. 3:**
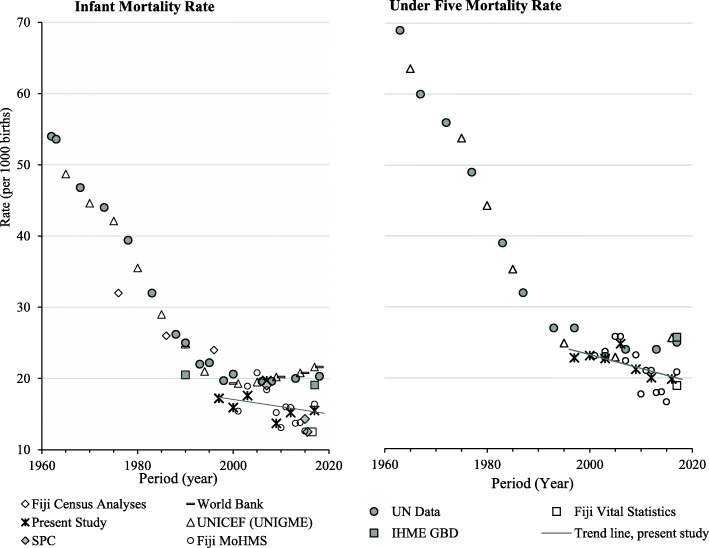
Published Infant Mortality Rates and Under 5 Mortality Rates for Fiji 1960–2018. Fiji Census Analysis [6, 31, 32]; World Bank [33]; UN Data [34]; Fiji Vital Statistics [16]; UNICEF (UNIGME) [35]; IHME GBD (Institute for Health Metrics and Evaluation) [36]; Fiji MoHMS [37, 38]; SPC (Pacific Community) [39].

**Fig. 4:**
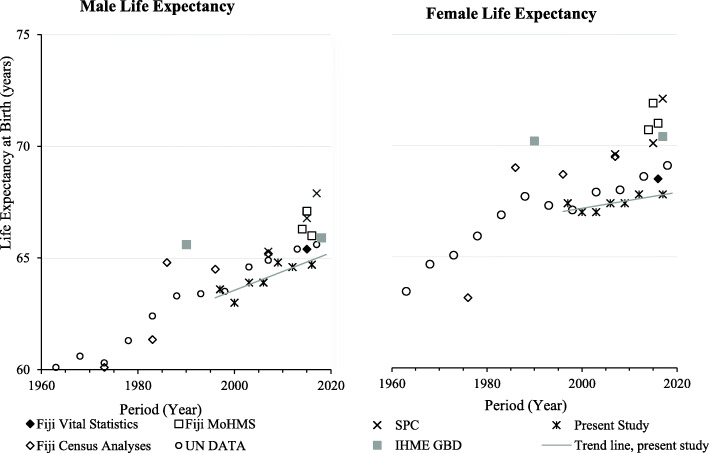
Published estimates for life expectancy by sex, Fiji 1960–2018. Fiji Vital Statistics [16]; Fiji Census Analyses [6, 31, 32]; Fiji MoHMS [37, 38]; UN Data [34]; IHME GBD (Institute for Health Metrics and Evaluation) [36]; SPC (Pacific Community) [39].

Published data from various sources show a plateau in IMR and U5M from 1995 (Fig. 3). The empirically based estimates in this study show lower levels than agency estimates from 2010, coinciding with exclusion of stillbirths from < 1-year deaths. MoHMS annual reports show even lower IMRs, possibly affected by late recording of deaths. Published agency trends of LE (at birth) by sex are higher than from empirical data in this study (Fig. 4). The Fiji MoHMS Annual Reports for 2014–16 [37, 38] estimate LE for males at 66.3–67.1 years; and for females 70.7–71.0. years. These estimates are higher, based on comparison of 95% CIs, than the LE estimates calculated in this study for 2014–17 (Table 3). The Fiji Vital Statistics Report estimates of LE at birth for 2015–17 (men 65.4; women 68.5) [16] are only slightly higher than LE estimates calculated here (2014–17). GBD estimates of LE increases in Fiji over 1995–2017 were limited to around 1 year, with estimates of male LE 65.9 and female LE 70.4 years in 2017, [40], compared to 64.6–64.9 and 67.6–68.0 years estimated in this study (Table 3). Analysis of LE in Fiji from FBoS is one of the few sources to report measures by ethnicity. Following the 2007 census, estimates of LE at birth for males was: 65.2 years for i-Taukei and 64.9 years for Indians; and for females: 67.4 years for i-Taukei and 72.2 years for Indians [17, 26]. The FBoS-reported LE’s show a similar pattern to that found in the current study, where average LE at birth increased progressively across the four groups (in order): Indian males, i-Taukei males, i-Taukei females and finally Indian females, who had the highest LE.

## Discussion

Previous studies reported declines in IMR from 1976 to 1996–98, with larger declines in Indians compared with i-Taukei, resulting in similar IMRs for both ethnicities by 1996–98 [3]. In this study, the IMR decline continued over 1996–2017, with an interruption during 2002–07, and stability since 2011. Changes in foetal death-recording practices and electronic mortality recording systems may have contributed to inclusion of foetal deaths in mortality data and artefactually elevated IMRs reported elsewhere. During 2000–2008 the stillbirth form was not in use, with stillbirths identified on a new MCCD only after 2009. The IMR reported by the MoHMS in 2009 noted that infant deaths include some stillbirths, [37] also impacting reported trends in U5M.

IMRs published in MoHMS Annual Health Status Reports (HSR) from 2001 to 2015 [37] are generally lower than estimated in this analysis. Since live births are consistent with those in this study, differences are likely due to under-enumeration of infant deaths in the MoHMS HSR from late recording of deaths, after publication of annual reports. The lower values of IMR and U5M, used as baseline measures in setting targets for IMR and U5M by 2020 in the MoHMS National Strategic Plan 2016–20, [41] underestimate the reduction in infant deaths required. For 2006–08, the FBoS 2007 Census Analytical Report [26] provides aggregated deaths (18,984) identical to the present study prior to the exclusion of 54 stillbirths (ICD10 code P95). Higher IMRs reported by FBoS for 2006–08 can be attributed to inclusion of stillbirths.

For young adult men (15–34 years), the probability of death for i-Taukei was similar to Indians in 1996–98, but lower in i-Taukei by 2014–17 compared to Indians (*p* < 0.05), due to declines for i-Taukei (*p* < 0.05). For young adult women, the probability of death remained low, and similar for i-Taukei and Indians from 1996 to 98 to 2014–17, however decomposition of the LE gap highlights higher mortality at 30–34-years for i-Taukei offset by higher mortality at 15–19-years for Indians. The cause of death structure of these differences requires investigation. Trends in mid-age adult mortality over 1996–2017 differ by sex and ethnicity; reporting mortality only for the total population by sex, rather than by sex and ethnicity, obscures these divergences. Explanations for differing mortality trends by sex and ethnicity in the 35–59 age group may reflect risk factor prevalence trends [42].

Decomposition of LE gaps between 1996 and 98 and 2014–17 shows differing age contributions by sex and ethnicity. Decomposition of the LE gap by cause of death will further inform contributions to these LE gaps.

The LE plateau previously reported for both ethnicities and sexes in Fiji, based on analysis of death records to 2008 [3] has continued to 2017. For the total male population, LE increased more than in each ethnic group individually; the increase can be partially attributed to i-Taukei, with a higher LE, making up a larger proportion of the total male population in 2017 compared to 1996. The MoHMS Annual Health Status reports (2014 to 2016) do not describe the method for calculating LE [37]. However, the 2014 report includes an estimated population of 933,204, [37] based on projected average intercensal annual population increases of 1.6% [25] between 2007 and 2017, greater than the actual average annual growth rate of 0.6% [5]. The use of these higher population estimates for calculating mortality rates would partly account for the higher LE estimates than in the present analysis. Late registration of deaths is noted as a limitation in the Vital Statistics Report, [16] with 6992 deaths recorded for 2017, compared with 7403 deaths here, due to late death registrations. This demonstrates a source of considerable inaccuracy that can arise from premature use of death data which is under-registered.

The 2017 GBD study identified Fiji as one of 20 countries where male LE has increased by less than 10 years since 1950 [40]. While LE estimated in this study from empirical death data are lower than estimated by GBD, both sources show stagnation in LE over recent decades. GBD classifies Fiji as a middle Socio-Demographic Index (SDI) country, and in 2017 the average LE in middle SDI countries was 71.1 (males) and 77.4 (females), considerably higher than LE estimates for Fiji from both GBD and this empirical study. The increases in SDI in Fiji do not appear to have translated into parallel increases in LE.

The Mortality Transition in Fiji Report, published by FBoS following detailed analysis of the 2007 census, also reports stagnation in LE beginning in the 1980’s and continuing until 2007 [17]. This report suggests possible contributors to this outcome: economic factors, including unemployment and emigration of healthy, working age people following the instability of three political coups in 1987, 2000 and 2006, and an increase in “lifestyle disease” [17]. The MoHMS National Strategic Plan 2016–2020 targets significantly increasing the number of nurses, doctors and other health workers [41]. The increasing burden of health care needs created as populations age, with increasing levels of chronic disease, can be expected to further constrain the health care system’s ability to meet the health needs of the community, potentially contributing to increased morbidity and mortality.

According to the MoHMS National Strategic Plan 2016–2020, approximately 78% of all deaths and 40% of deaths < 60 years are due to NCDs [41]. The top three causes of mortality as reported by the MoHMS remain diseases of the circulatory system, endocrine and metabolic conditions (mostly diabetes), and neoplasms [37, 38]. Significant increases in the prevalence of high blood pressure in both sexes and both ethnicities occurred between 1980 and 2011 [11]. T2DM prevalence [12] and incidence [13] also increased in Fiji between 1980 and 2011. Obesity rates, using standard BMI cut-off points, are higher in i-Taukei than Indians, and higher in women than men, but T2DM prevalence is higher in Indians than i-Taukei [12]. Tobacco smoking in both sexes and ethnicities decreased over 1980–2011, with most of the reduction occurring before 2000 for i-Taukei men, after which prevalence formed a plateau; whilst for Indian men the decline continued until 2011 [43]. Mortality and LE trends from cardiovascular disease, T2DM and lung cancer are the result of cumulated exposure to risk factors over previous decades; and based on reported trends in risk factors the current plateau in LE is expected to continue.

This analysis utilises more complete primary data than previously employed for published estimates of mortality and LE in Fiji and uses 2017 Fiji census data as denominators, which enumerated a smaller population than previously projected. Exclusion of foetal deaths, inclusion of late reported infant and other deaths, and removal of duplicate records, allows calculation of IMR, as well as total mortality and LE, from more accurate primary data.

Limitations in the present analysis include lack of birth data by sex, missing age from some death records and lack of ethnicity data for the 2017 census. IMR and U5M were calculated by ethnicity but not by sex. Divergent trends in mortality in the < 5-year age group by sex are evident from deaths and census populations < 5-years, used in life tables. Decomposition analysis of LE gaps suggests that the improvements seen in U5M can be attributed to reductions in U5M for females, both i-Taukei and Indian, while little improvement is evident in U5M for males and comparison shows that higher U5M for i-Taukei, both male and female, contributes negatively to the LE gap between the ethnic groups. The results are also affected by an unusually high number of i-Taukei male infant deaths in 2014–17 for reasons that are not obvious. Births by sex should be routinely collated to allow calculation of IMR and U5M by sex as well as by ethnicity. Contributions of causes of death to these differences should be investigated. The small number of deaths with unknown age were redistributed proportionately prior to analysis, which is an approximation, and may not reflect that age is more likely to be missing in older descendants, and varies between years; however, is superior to some other reports [23] which appear to exclude incomplete records.

Populations by ethnicity were not published from the 2017 census. Between the 1996 and 2007 censuses the FBoS used the 1996 census as a base population and then continuously added births and immigration, and subtracted deaths and emigration, to maintain updated population statistics by ethnicity. These calculations were found to be in close agreement with the 2007 census population [26]. Populations by ethnicity for 2017 in this study were estimated by utilising ethnic-specific population projections published by the FBoS in 2017, [7] and the 2017 census data by sex and 5-year age groups. The ethnicity of the decedent was identified on the death certificate and may have been provided by the individual on hospital admission or by a family member. Differing sources of ethnicity between the death (numerator) and population (denominator) may bias results. Differences by ethnicity have been found to be an important explanatory variable in epidemiological, demographic and socio-economic analyses in Fiji, including in health-related studies [26].

## Conclusions

IMRs reported in this study are higher than recent MoHMS estimates, and lower than those reported by international agencies, but are likely to be more accurate because of inclusion of more complete death records and exclusion of stillbirths. The reduction in young adult (15–34 years) male mortality can be attributed to reduction among i-Taukei, concealing the stagnation in mortality among Indians. The reduction in mid-age (35–59 years) mortality for men, due to reduction in Indians, conceals a stagnation in i-Taukei. Relatively unchanged mid-age mortality for women, conceals the mortality increase in i-Taukei women. Analysis following such stratifications improves understanding of the epidemiology of mortality in the whole population. The small increase in LE (at birth) in both sexes and ethnicities over 1996–2017 is significantly less than might be expected in a middle SDI country with LE’s in 1996–98 starting from a low base at 62–68 years. LE reported in this study is lower than reported by Fiji national agencies, and by several international agencies. Under-enumeration of deaths, inflated population estimates (projections from previous censuses), and incorrect modelling assumptions likely contribute to these discrepancies.

## Data Availability

The mortality dataset analysed is not publicly available but may be requested by submission of a Data Request form to the Data Analysis Management Unit (DAMU) of the Ministry of Health and Medical Services, Fiji. The non-confidential information used in this publication, was compiled in accordance with the Information Act of 2018 of the Republic of Fiji but which DAMU has no authority to independently verify. The Data Analysis Management Unit of the Ministry of Health and Medical Services cannot and does not represent that the data was appropriate for this publication, or endorse or support any conclusions that may be drawn from the use of the data.

## Abbreviations

BDM: Births, Deaths and Marriages
BMI: Body mass index
CI: Confidence interval
DAMU: Data Analysis Management Unit
DASR: Directly age-standardised death rate/s
DoB: Date of birth
DoD: Date of death
FBoS: Fiji Bureau of Statistics
GBD: Global Burden of Disease Study
ICD: International Classification of Diseases
IHME: Institute for Health Metrics and Evaluation
IMR: Infant mortality rate
LE: Life expectancy
MCCD: Medical Certificate of Cause of Death
MoHMS: Ministry of Health & Medical Services
NCDs: Non- communicable diseases
NoB: Notification of birth
PICs: Pacific Island Countries
SDG: Sustainable Development Goal
SDI: Socio-Demographic Index
SPC: Pacific Community
T2DM: Type 2 diabetes mellitus
U5M: Under-five mortality
UN: United Nations
UNICEF: United Nations Children’s Fund
UNIGME: United Nations Inter-agency Group for Child Mortality Estimation
WHO: World Health Organisation
